# Connecting the Dots: Structural Racism, Intersectionality, and Cardiovascular Health Outcomes for African, Caribbean, and Black Mothers

**DOI:** 10.1089/heq.2021.0077

**Published:** 2022-06-07

**Authors:** Deborah Baiden, Monica Parry, Kara Nerenberg, Edith M. Hillan, Maman Joyce Dogba

**Affiliations:** ^1^ Lawrence S. Bloomberg Faculty of Nursing, University of Toronto, Toronto, Canada.; ^2^ Department of Medicine, Obstetrics & Gynecology, Community Health Sciences, University of Calgary, Calgary, Canada.; ^3^ Department of Family and Emergency Medicine, Université Laval, Quebec, Canada.

**Keywords:** cardiovascular health, health equity, intersectionality, structural racism, women

## Abstract

African, Caribbean, and Black (ACB) women globally experience health inequities that impact on their cardiovascular health outcomes during the perinatal period, and for years after. Aside from being at a high risk of having and dying from hypertensive disorders of pregnancy, ACB women who survive face a lifelong risk of cardiovascular disease years after the diagnosis. Racism as a determinant of health intersects with gender, societal structures, and immigration status to contribute to cardiovascular health and access to quality health care services for ACB women. Equitable policies and culturally appropriate programs are needed to improve the cardiovascular health of ACB women.

## Introduction

On October 22, 2020, Dr. Chaniece Wallace, an African American pediatric resident, died from the complications of preeclampsia, a hypertensive disorder of pregnancy (HDP), days after the birth of her first child.^1^ Black women in the United States die at a rate of 40.8 pregnancy-related deaths per 100,000 live births between 2007 and 2016 in comparison with the national rate of 16.7 pregnancy-related deaths per 100,000 live births.^2^ Many Black women die from pregnancy-related cardiovascular diseases including cardiomyopathy and HDP.^2^ There is evidence to show that women of African and Caribbean descent living in Australia, Canada, France, Spain, Sweden, and the United States are more likely to experience severe preeclampsia than women of other ethnicities/races and to die from it.^3–5^

This shows the global health inequities in this population. Furthermore, emerging evidence suggests that women who experience any HDP (gestational hypertension, preeclampsia, etc.) have higher future cardiovascular disease risks than women who are normotensive during pregnancy.^6^ Women with a history of HDP have a long-term risk of several cardiovascular diseases: cardiac failure, coronary heart disease, stroke, and ultimately death.^6^ Thus, African, Caribbean, and Black (ACB) women are likely to experience lifelong risks of cardiovascular disease after HDP.

Dr. Wallace was highly educated, employed as a health care practitioner, had access to health care, and had a supportive family. These privileges are protective factors that are traditionally associated with reduced cardiovascular disease risks.^7^ Although advanced education and being employed in a good paying job often signify a higher socioeconomic status, we hypothesize that these factors alone do not appear adequate to protect ACB mothers from dying due to pregnancy-related cardiovascular complications. Black women with postsecondary education or higher are 5.2 times more likely to die from pregnancy-related cardiovascular complications than their White peers.^2^ This finding suggests that individual level factors are only one contributor to the health inequities experienced by ACB mothers.

Kimberle Crenshaw asserts in her theory of intersectionality that people experience injustices uniquely based on the overlapping of their positions in multiple social constructs.^8^ This highlights that system level factors may play a significant role in cardiovascular mortality and morbidity rates among these mothers. Emerging data have highlighted the importance of structural racism's long-lasting effects impact on Black mothers through generations.^9^ There are two main objectives of this article, the first is to provide a critical discussion linking structural racism to cardiovascular health outcomes for ACB mothers. The second is to analyze the role of intersectionality in perpetuating pregnancy and cardiovascular health inequities among ACB mothers.

## Structural Racism and Cardiovascular Health Outcomes for ACB Mothers

Racism is a key determinant of health and wellness and is ingrained in socially constructed structures that further increase health inequities of populations because of their race.^10^ Structural racism is defined as “the totality of ways in which societies foster racial discrimination, through mutually reinforcing inequitable systems…that in turn reinforce discriminatory beliefs, values, and distribution of resources, which together affect the risk of adverse health outcomes.”^11^ Thus, structural racism is broad, interlinked with various systems, and multilayered.

Racism is an established determinant of cardiovascular health outcomes.^12^ Black women in the United States face racism daily and experience a more gendered form of racism: “racialized pregnancy stigma” (p. 487), during the perinatal period, and both inside and outside the health care system.^13^ The experience of racism during the perinatal period causes a “ripple effect” that hinders Black mothers' engagement with the health care system, and their social support-seeking behaviors.^13^ This may result in an added source of stress during the perinatal period that may translate into missed prenatal appointments whereby risk factors for cardiovascular morbidity and mortality during and after pregnancy are not detected and/or managed through standard evidence-based preventive interventions.

Continued racism precipitates stress, which is a risk factor for hypertension and cardiovascular disease, including left ventricular hypertrophy and cardiac failure.^12^ Although pregnancy has been referred to as a natural stress test for women's cardiovascular health, for ACB women, racism compounds this physiological stress. Thus, the intersectionality of these factors may account for the high incidence of HDP in this population. As a result of this systemic racism, populations of color (i.e., ACB people) often receive substandard care during pregnancy, which may be further exacerbated by unconscious biases from health care providers.^14^

In addition, Black mothers' concerns are often dismissed by health care professionals resulting in increased mortality rates.^15^ Other examples of systemic racism including police brutality profoundly increase stress in Black pregnant women.^16^ Black women living in communities with excessive police force are more likely to experience cardiovascular disease than White women.^17^ Cardiovascular diseases are associated with racism, with post-traumatic stress disorder markedly explaining this relationship.^18^

## Intersectionality and Cardiovascular Health Outcomes for ACB Mothers

It is important to note that ACB mothers share several social identities that intersect to impact their individual cardiovascular health. Social identities of race, ethnicity, gender, and socioeconomic status intersect to place Black women with a low-income level at a higher risk of cardiovascular diseases.^19^ Gender as a social identity perpetuates inequalities in cardiovascular health for women.^7^ Black women come up against race-specific gender stereotypes during the maternal period and beyond, which may limit access to health care and support services, ultimately affecting their health and well-being.^13^

Black women born outside the United States (e.g., Sub-Saharan Africa, the Caribbean, and other regions) had a lower risk of preeclampsia than Black women born in the United States.^20^ Several factors can be attributed to the variations among these groups despite sharing the same racial identity. Cultural norms followed by ACB women born outside the United States may partly explain the variance between those born outside the United States and those born within the United States.^20^

Despite the shared racial identity, culture intersects with race and other identities uniquely for ACB mothers' cardiovascular health, depending on their place of origin. Black immigrant women who had lived in the United States for >10 years had a higher risk of preeclampsia than Black newcomer women.^20^ There are reasons that could account for these differences. First, the process of settling in the United States leads to ACB immigrant women integrating and adapting their culture, including dietary preferences.^20^

Second, immigration procedures are designed to grant residency only to fit and healthy immigrants, thus accounting for the differences in preeclampsia risk.^20^ The social identities of race, ethnicity, gender, and immigration status intersect to impact cardiovascular health outcomes of ACB mothers. Furthermore, factoring in evidence about the impact of structural racism on cardiovascular health outcomes of ACB mothers heightens the connection between structural racism and the intersection of race, gender, and immigration status.

The United Nations declared 2015–2024 as the International Decade for People of African Descent and has also put forth 17 sustainable development goals as part of the 2030 Agenda for Sustainable Development.^21,22^ In relation to cardiovascular health of ACB mothers, the specific sustainable goals that could improve the health and well-being of ACB mothers are goals of good health and well-being (goal 3), gender equality (goal 5), reduced inequalities (goal 10), and partnerships for the goals (goal 17).^22^

Health care professionals should engage in health promotion among ACB mothers with consideration of the inequalities of gendered racism, structural barriers, and the need to center the voices of ACB mothers for empowerment and effective partnerships to reduce rates of HDP and improve the cardiovascular health of this population. There is a need for advocacy for broad collaborative strategies involving Black mothers, family, doulas, religious organizations, and integrated teams of health professionals to improve cardiovascular health outcomes for Black mothers.^14^

## Conclusion and Call to Action

There is an urgent need for policymakers at all levels of government to consider intersectionality in the policy-making process. This is because legislation and policies impact populations differently, based on their socially constructed identities. An intersectionality-informed policy should be representative of the multidimensionality of the population it seeks to impact, at the decision-making table, and as guiding principles. In addition, health care professionals need to provide personalized health services tailored to the needs of ACB mothers. Health care professionals must engage in reflexive practice in their care.

Curricula for the training and education of health care and social services professionals must be embedded with antiracism and cultural competency. ACB women should actively be involved in the codesign and implementation of health promotion strategies for better cardiovascular health during the perinatal period and beyond. Furthermore, health researchers and scientists should engage diverse populations in their research work to ensure a true representation of the world in which we live.

The United Nations' Sustainable Development Goals could serve as a framework through which health researchers worldwide can work together to achieve better health outcomes for everyone. In conclusion, health equity can only be truly achieved globally, when no one is left behind such as Kira Johnson, a pilot, marathon runner, and healthy Black mother who died in April 2016, hours after her second son's birth.^23^

## Abbreviations Used

ACB: African, Caribbean, and Black
HDP: hypertensive disorder of pregnancy
